# Prediction of rifampicin resistance beyond the RRDR using structure-based machine learning approaches

**DOI:** 10.1038/s41598-020-74648-y

**Published:** 2020-10-22

**Authors:** Stephanie Portelli, Yoochan Myung, Nicholas Furnham, Sundeep Chaitanya Vedithi, Douglas E. V. Pires, David B. Ascher

**Affiliations:** 1 Department of Biochemistry and Molecular Biology, Bio21 Institute, University of Melbourne, Victoria, 3010 Australia; 2 Computational Biology and Clinical Informatics, Baker Heart and Diabetes Institute, Melbourne, 3004 VIC Australia; 3 Department of Infection Biology, London School of Hygiene and Tropical Medicine, Keppel Street, London, WC1E 7HT UK; 4 Department of Biochemistry, University of Cambridge, Cambridge, UK; 5 School of Computing and Information Systems, University of Melbourne, Victoria, 3010 Australia

**Keywords:** Computational models, Protein analysis

## Abstract

Rifampicin resistance is a major therapeutic challenge, particularly in tuberculosis, leprosy, *P. aeruginosa* and *S. aureus* infections, where it develops via missense mutations in gene *rpoB.* Previously we have highlighted that these mutations reduce protein affinities within the RNA polymerase complex, subsequently reducing nucleic acid affinity. Here, we have used these insights to develop a computational rifampicin resistance predictor capable of identifying resistant mutations even outside the well-defined rifampicin resistance determining region (RRDR), using clinical *M. tuberculosis* sequencing information. Our tool successfully identified up to 90.9% of *M. tuberculosis rpoB* variants correctly, with sensitivity of 92.2%, specificity of 83.6% and MCC of 0.69, outperforming the current gold-standard GeneXpert-MTB/RIF. We show our model can be translated to other clinically relevant organisms: *M. leprae*, *P. aeruginosa* and *S. aureus*, despite weak sequence identity. Our method was implemented as an interactive tool, SUSPECT-RIF (StrUctural Susceptibility PrEdiCTion for RIFampicin), freely available at https://biosig.unimelb.edu.au/suspect_rif/.

## Introduction

The drug rifampicin (Rif) was initially developed in the 1960s, through modification of the natural isolate rifamycin B^1^. This novel, orally available, semi-synthetic agent, was shown to block the DNA-dependent RNA polymerase transcription initiation complex^1^, which confers its bactericidal activity. Since its discovery, Rif has become part of the backbone treatment for mycobacterial tuberculosis (TB) and leprosy infections and remains their most effective therapeutic available today^2,3^. The introduction of Rif to the TB multi-drug regimen reduced treatment time from 18 to 9 months^4^, which was further shortened to 6 months through introduction of pyrazinamide^5^. Inclusion of Rif as part of the multi-drug therapy regimen for leprosy in the mid-80 s reduced initial disease incidence from over 5 million to less than 200,000 cases in the two following decades^6^. Clinically, Rif is also reserved as a last line drug in multi-drug resistant (MDR) infections from *Staphylococcus aureus* (methicillin-resistant *S. aureus*; MRSA)^7^ and *Pseudomonas aeruginosa*^8^, among other infections^7^.

Following its introduction, resistance in the mycobacteria *M. tuberculosis* and *M. leprae* has developed as a direct result of evolutionary purging upon extended Rif exposure, primarily through missense mutations in drug targets or activating enzymes^9^. This phenomenon affected over half a million tuberculosis cases in 2018, of which, 78% were also classified as MDR-TB^2^. Similarly, according to global surveillance efforts in 2018, Rif resistance is observed in 3.8% of all leprosy cases, having a higher incidence (5.1%) upon relapse (secondary resistance)^3^. Resistance occurs primarily through the accumulation of missense mutations within the *rpoB*^7,10^ gene, which encodes the RNA polymerase β-subunit. A specific 81 bp region within this gene, known as the rifampicin resistance determining region (RRDR), has been widely associated with drug resistance^10^. This region is highly conserved amongst species, as it forms part of the transcription cleft and active site.

The current WHO-endorsed test for identifying Rif resistance in TB, the GeneXpert-MTB/RIF, is a molecular test which solely focuses on identifying variants in the RRDR^2,11^. According to published reports, this test can detect Rif resistance with a sensitivity of 95% and specificity of 98–99%^12,13^ in both pulmonary^13^ and extra-pulmonary^12^ TB. Systematic evaluations of resistance mutations within *rpoB* suggest, however, that performance is much lower clinically, with estimates that a third of resistant TB infections are missed^14^. In particular, different studies have identified resistance-associated mutations outside the RRDR in the *Mycobacterium tuberculosis* (*Mtb*) *rpoB* gene^15–17^, which are consequently misdiagnosed as susceptible by the gold standard test.

In leprosy, resistance to Rif was traditionally identified through the mouse footpad model, which takes months to culture and requires specialised personnel^3,18^. This might account for the lower incidence of Rif resistance reported in leprosy when compared to TB. Advancements in DNA sequencing and PCR have enabled for a prompt identification of resistance biomarkers within *rpoB*^3^. These molecular techniques have been used by the WHO Global Leprosy Programme, where they also focused on the RRDR in *M. leprae* to identify resistance mutations^3^. Results from these tests were at 100% sensitivity and 99% specificity in detecting resistance mutations^3^, but again only focused on a small region across the whole gene.

The broad distribution of mutations across the gene in *Mtb* might indicate alternative Rif resistance mechanisms beyond interference with drug binding. In our earlier work, we investigated the potential mechanisms of rifampicin resistant mutations across the whole *Mtb* gene and found that disruptions in protein–protein interactions, leading to destabilisation of the RNA polymerase complex and nucleic acid affinity^19^, are important contributing molecular factors. It is well established that mutations within the *rpoC* gene^20,21^ compensate disruptive effects of resistance-causing *rpoB* mutations, which explains an overall normal transcriptional function despite a loss of intermolecular affinities in resistant bacteria. These intricate mechanisms at the protein complex level cannot be encompassed by the current molecular tools being used, which focus only on the RRDR sequence. Further to this, whole genome sequencing techniques, despite their efficiency, do not explain how resistance arises at the molecular level—limiting them to the characterisation of known mutations, without any predictive capability for novel variations.

To overcome these limitations, we have used a computational approach to further our understanding of the molecular mechanisms leading to resistance and to build a novel, web-based diagnostic tool, SUSPECT-RIF (StrUctural Susceptibility PrEdiCTion for RIFampicin), to accurately and pre-emptively identify resistance mutations. Our structure-based diagnostic tool follows previous, clinically successful approaches in predicting TB drug resistance^22–25^. Despite only being trained on information readily available for *Mtb rpoB* mutations, we show that our tool is also effective in identifying resistant mutations in infections caused by *M. leprae, S. aureus* and *P. aeruginosa*.

In this work, we have computationally measured effects of missense mutations in *Mtb rpoB* on protein stability^26–28^, dynamics^29,30^, and interactions with Rif^31^, other proteins^26,32^, nucleic acids^26,33^, and metal ions^34^. Machine learning was used on these measurements to train, test and validate a Rif-resistance classifier as a novel diagnostic predictor. Our final tool, SUSPECT-RIF, incorporates sequence- and structure-based features to model how missense mutations lead to resistance.

## Results

**Figure 1:**
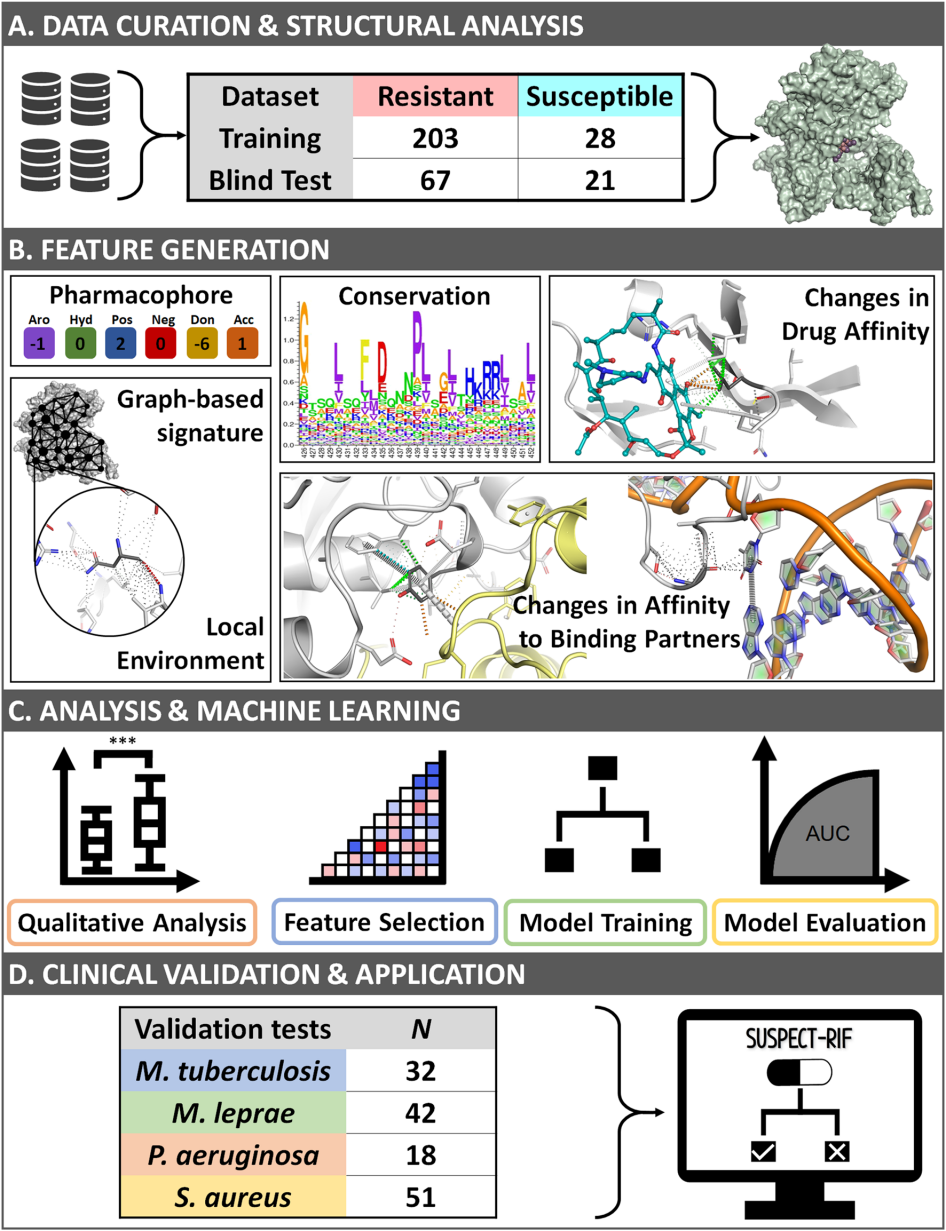
Overview of SUSPECT-RIF workflow. Mutational data was manually curated from the literature, followed by structural analysis to ensure high data quality (**A**). Structural and Sequence-based properties were calculated and their ability to distinguish between mutation classes was assessed (**B**). Evolutionary and biological insights were used to train a machine learning classifier to accurately identify novel resistant and susceptible variants (**C**). Evaluation of the final model across blind clinical test sets from *M. tuberculosis* showed high performance and accurately identified clinical resistance variants from *M. leprae*, *P. aeruginosa* and *S. aureus* (**D**). SUSPECT-RIF is available at https://biosig.unimelb.edu.au/suspect_rif.

The general methodology of this project is summarized in Fig. 1 and is divided into four main phases. The initial phase of this project sought to combine the current biological understanding of Rpob function and Rif resistance in the literature, with structural and mutational information. Notably, a thorough quality check of the crystallographic structure^35^ and mutational information^15^ was carried out to ensure that the final structure-based predictor was built on biologically accurate data (Fig. 1A). Our training set contained 203 resistant and 28 susceptible mutations obtained from the London School of Hygiene and Tropical Medicine^15^. An independent test set was curated from online databases^36–38^ and contained 67 resistant and 21 susceptible mutations (Suppl. Figure 1). In the feature generation phase (Fig. 1B), different molecular effects of mutations were predicted using available tools. Additionally, features describing conservation^39–44^, the mutational local^45–48^ and global environments^49,50^ were also calculated. Here, features describing “local environments” accounted for protein properties at the mutation site prior to (e.g. residue depth) and after mutation (e.g. changes in protein stability^26–28^). The “global environment” was calculated through graph-based signatures^49,50^, which capture the overall protein as a series of local “nodes” connected to the mutation site “node” across different distance patterns. Further information on the features used is detailed in Methods. Next, a qualitative analysis was carried out to better understand the underlying resistance mutation mechanisms at the protein level, by using local environment and affinity change measurements (Fig. 1C). During machine learning, all features were used to train and test different classification algorithms for comparison and evaluation. This process included optimisation strategies such as feature selection. The best performing algorithm was selected and validated through independent clinical tests (Fig. 1D) prior to the final implementation phase. Here, we describe how this methodology was used to develop a predictive classifier for Rif resistance, demonstrating application across resistant mutations in four distinct organisms where Rif is used: *M. tuberculosis*^36–38,51,52^, *M. leprae*^53,54^, *P. aeruginosa*^55^ and *S. aureus*^56^.

### Qualitative structural analysis

**Figure 2:**
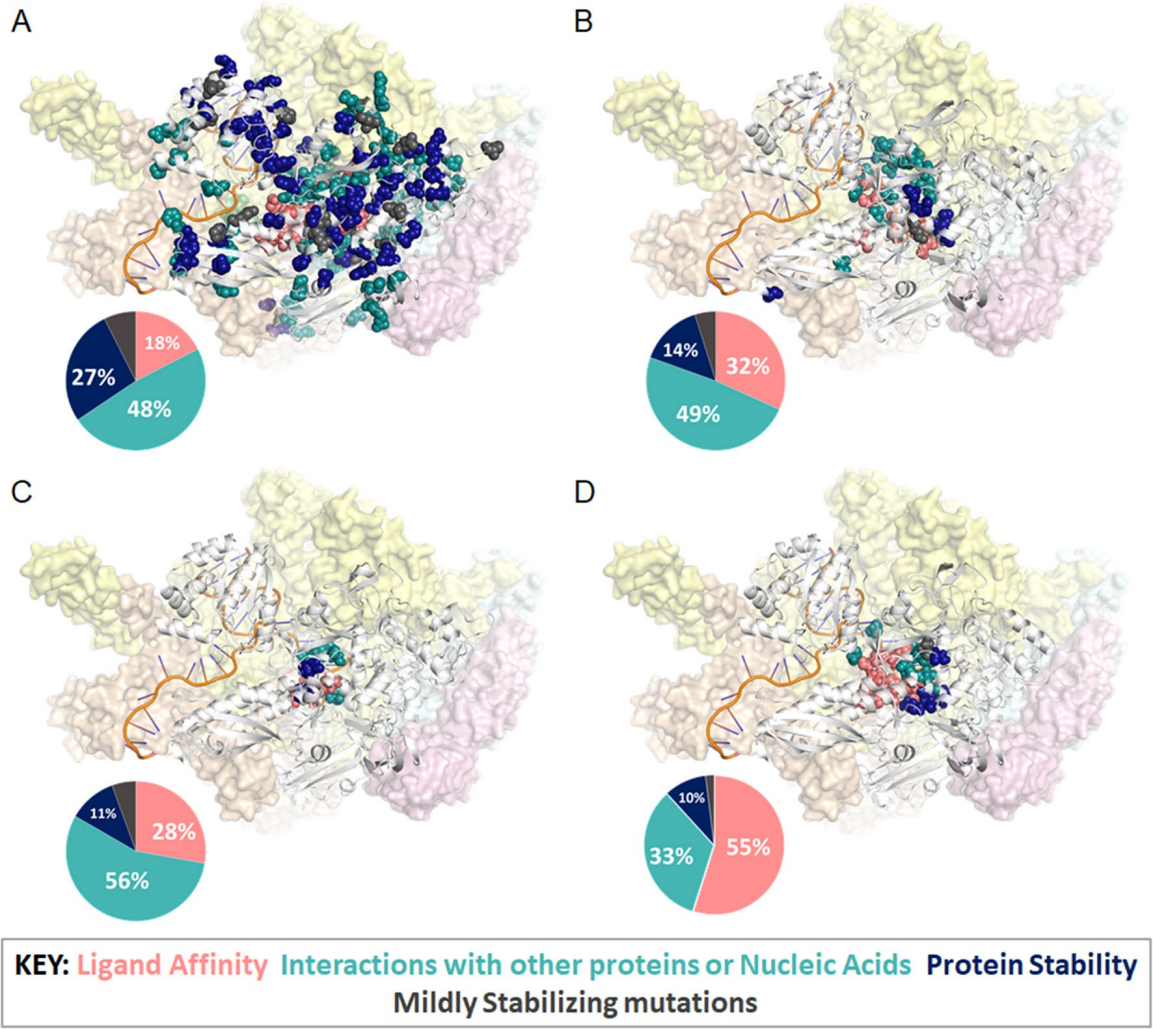
Graphical representation of mutations present within our study for (**A**) Resistant *M. tuberculosis*, (**B**) Resistant *M. leprae*, (**C**), Resistant *P. aeruginosa*, and (**D**) Resistant *S. aureus* datasets. Mutations labelled according to predominant effect: ligand affinity (salmon), non-ligand affinities (teal), protein stability (blue) and mildly stabilizing mutations (grey).

A detailed qualitative analysis on RpoB, limited to resistance mutations, has already been published as part of a larger study exploring three different drug targets in TB treatment^19^. Here, we expanded our analysis to include susceptible mutations, a more comprehensive set of *Mtb* Rif-resistant mutations, and mutations across all species tested. For *M. tuberculosis* mutations, in silico biophysical calculations were carried out on the experimental crystal structure of *Mtb* RNA polymerase complex (PDB id: 5UHC^35^). As no experimental structure of the other three organisms was available, we modelled these structures through comparative homology modelling using the *Mtb* structure^35^ as the template and used them for feature calculation. When considering Rif resistance mutations across all species tested, disruptions in protein–protein interactions^26^ and subsequent reduction in nucleic acid affinity^26^ were the most predominant mechanisms of resistance, followed by reductions in ligand affinity^31^ and protein stability^27^ (Fig. 2). This is in line with our previous qualitative analysis carried out on *M. tuberculosis* mutations alone^19^, and provided a good starting point for building a Rifampicin resistance predictor for these organisms.

When compared to *M. tuberculosis* (*n* = 270; Fig. 2A) resistance mutations, *M. leprae* mutations (*n* = 41; Fig. 2B) follow similar trends, where the major effects were as a result of protein–protein and protein-nucleic acid affinity decrease. This is possibly because of higher sequence identity (SI = 95%) amongst the mycobacterial RpoB proteins. According to our analysis, between the two mycobacteria, effects on protein stability were more pronounced for *Mtb* (27% of *Mtb* mutations vs. 14% of *M. leprae* mutations, *p*-value = 0.13), while those on ligand affinity were significantly more distinct in *M. leprae* mutations (17% of *Mtb* mutations vs. 32% of *M. leprae* mutations*, p*-value = 0.05). *P. aeruginosa* (*n* = 18; SI = 55%; Fig. 2C) also follows the same trend, although from the non-ligand interactions, disruption of nucleic acid affinity (50% of *P. aeruginosa* mutations) is stronger than for protein–protein affinity (6% of *P. aeruginosa* mutations). For *S. aureus*, however, (*n* = 51; SI = 61%; Fig. 2D) the main mechanism of resistance is through disruption of ligand affinity, which might shed light on resistance properties associated with multi-drug resistance in MRSA populations. Other possible causes underlying differences between non-mycobacteria and mycobacteria may be explained biologically through horizontal gene transfer.

## Feature analysis and engineering

Descriptive features were calculated to represent major biological functions and processes: protein interactions, local environment, graph-based signatures as measures of global environment, pharmacophore modelling, and conservation (Fig. 1). A total of 298 features were calculated.

**Figure 3:**
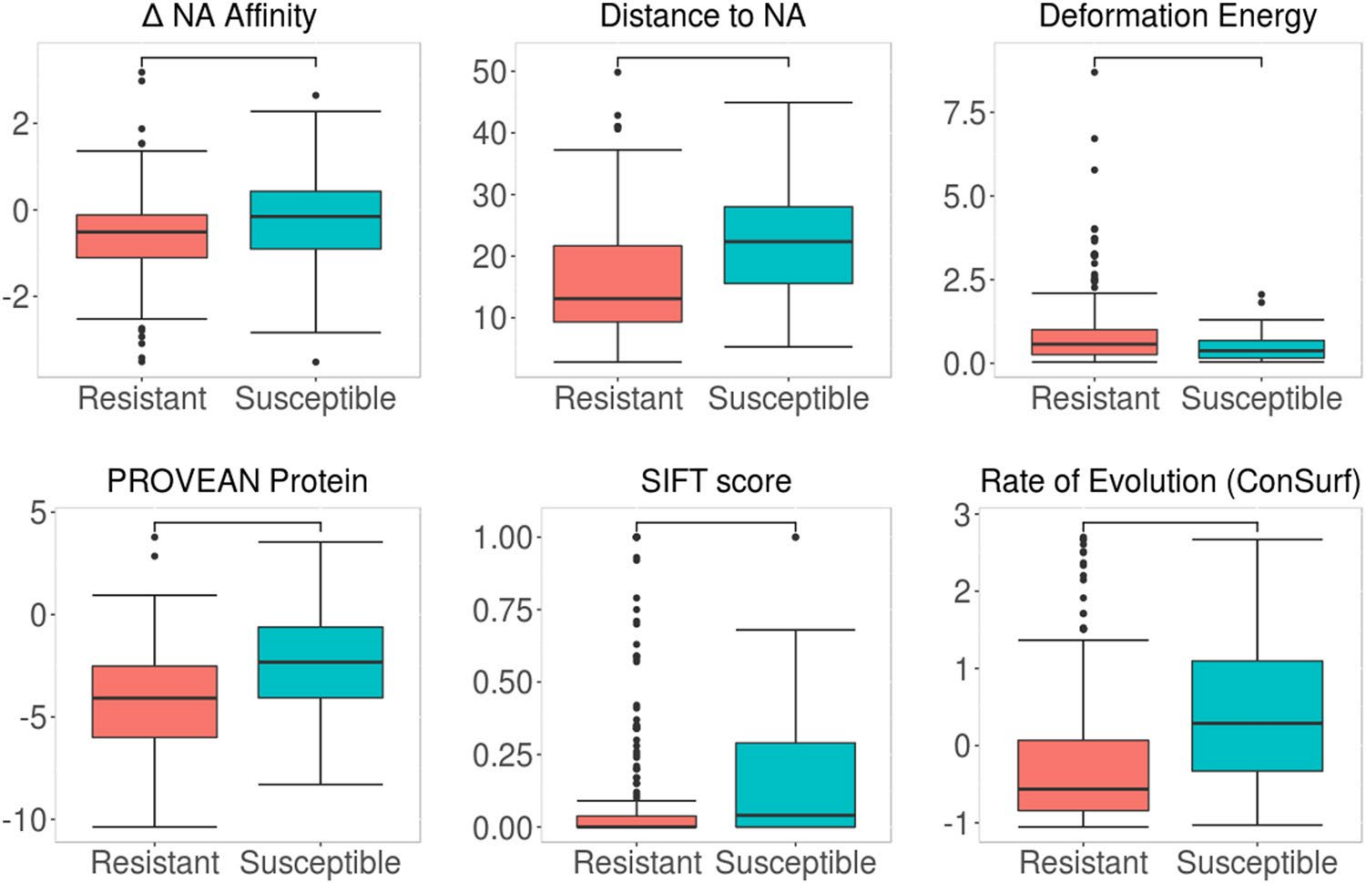
Comparison of the distribution of key properties between resistant and susceptible Mtb mutations. Selected properties included structure-based features (change in nucleic acid affinity, deformation energy and distance to nucleic acid) and sequence-based features (PROVEAN, SIFT and rate of evolution scores). Statistical significance calculated by Welch sample t-test.

Prior to machine learning, we subjected our *Mtb* dataset to a Welch’s t-test, in order to identify features that can distinguish between the two phenotype classes, resistant (*n* = 270) and susceptible (*n* = 49; Fig. 3 and Suppl. Figure 2). Among the most distinguishing features (given by a *p*-value of < 0.05) were changes in nucleic acid binding affinity^26^ (*p* < 0.05), distance to nucleic acid (*p* < 0.0001) and Rif (*p* < 0.0001) and protein flexibility, denoted as deformation energy^48^ (*p* < 0.001). These features highlight the importance of considering structural effects when analysing, and consequently predicting, mutational phenotypes. Other highly distinguishing properties were sequence-based features PROVEAN Protein^41^ (*p* < 0.0001) and SIFT^39,40^ (*p* < 0.05), which measure the effect of mutations on protein function based on sequence alignment^39–41^ and amino acid properties^39,40^, and the conservation feature “Rate of Evolution” given by ConSurf^43^ (*p* < 0.0001). Considering the highly conserved *rpoB* gene being studied, these results show that resistant mutations are more likely to cluster at highly conserved regions (given by a lower evolutionary rate from ConSurf) than susceptible ones. This clustering at conserved, prominent sites within the gene are thought to be crucial for their gain-of-function survival mechanism in the presence of a drug. As part of model optimisation, features chosen through greedy feature selection were also statistically compared through a correlation matrix (Suppl. Figure 3) in order to remove any redundancies within features.

This process removed the feature describing change in vibrational entropy as calculated by ENCoM^30^, as it correlated with the respectively calculated change in protein stability^30^.

Following removal of redundant features, bottom-up greedy feature selection was carried out, based on Matthews correlation coefficient (MCC). Our optimized model contained 41 features (Suppl. Table 1), which included representative features from the different classes considered including: graph-based signatures, stability effects, dynamics and flexibility measurements, pharmacophore changes and the changes in protein interactions with ligand rifampicin, nucleic acids and other proteins. Notably, while graph-based signatures provide a measure of global environment through different local environments at different distances from the mutation site, our feature selection only selected one of these features: Inter-HP:4.50, which accounts for hydrophobic and polar interactions within 4.50 Å of the mutational site. As only this feature from graph-based signatures was used, overall global effects are not represented in the final model, but rather, a physicochemical measure was added to the other local environment features.

**Table 1:** Predictive performance across training and non-redundant blind test sets.

Metric		Sensitivity	Specificity	MCC	F1 score	Accuracy	Precision
All	Training (n = 231)	89.7%	100%	0.72	0.90	90.9%	100%
	Test (n = 88)	100%	57.1%	0.71	0.91	89.8%	88.2%
Non-RRDR	Training (n = 193)	87.9%	100%	0.72	0.94	89.6%	100%
	Test (n = 39)	100%	76.2%	0.77	0.88	87.2%	78.3%

Numbers show performances throughout the whole gene, and outside of the rifampicin resistance determining region (RRDR). Performance on mutations within the RRDR was equivalent, with 100% sensitivity and 0% specificity (no susceptible mutations present).

The greedy feature selection approach used in this work is a heuristic approach, whereby the optimal combination of features is obtained in a stepwise, incremental manner. The final features obtained corresponded to our initial qualitative analysis results and are biologically relevant when considering overall interactions at the RNA polymerase cleft. To identify the contribution of each property feature to the final model, we trained a model under the same parameters on different subsets of features which likely work in synergy: conservation, intramolecular interactions, protein flexibility and dynamics, graph-based signature, ligand affinity, nucleic acid affinity, protein–protein interactions, physicochemical properties and presence in RRDR. MCC values depicting performance on the blind test for each subset model were compared (Suppl. Table 2). Notably, the major contributing feature for our final model was change in ligand binding affinity (MCC = 0.56). Other features contributed to the model through lower extents: physicochemical properties (MCC = 0.22), graph-based signature (MCC = 0.19), protein flexibility measurements (MCC = 0.17), changes in protein-nucleic acid interactions (MCC = 0.15), changes in protein–protein interactions within the RNA polymerase complex (MCC = 0.13) and conservation (MCC = 0.10). Finally, intramolecular interaction counts (MCC = 0.03) and presence in RRDR (MCC = 0.0) gave negligible contribution on their own, but are thought to enhance the effects of other features, e.g. affinity changes and changes in ligand affinity respectively when used within the final model.

## Model performance

**Figure 4:**
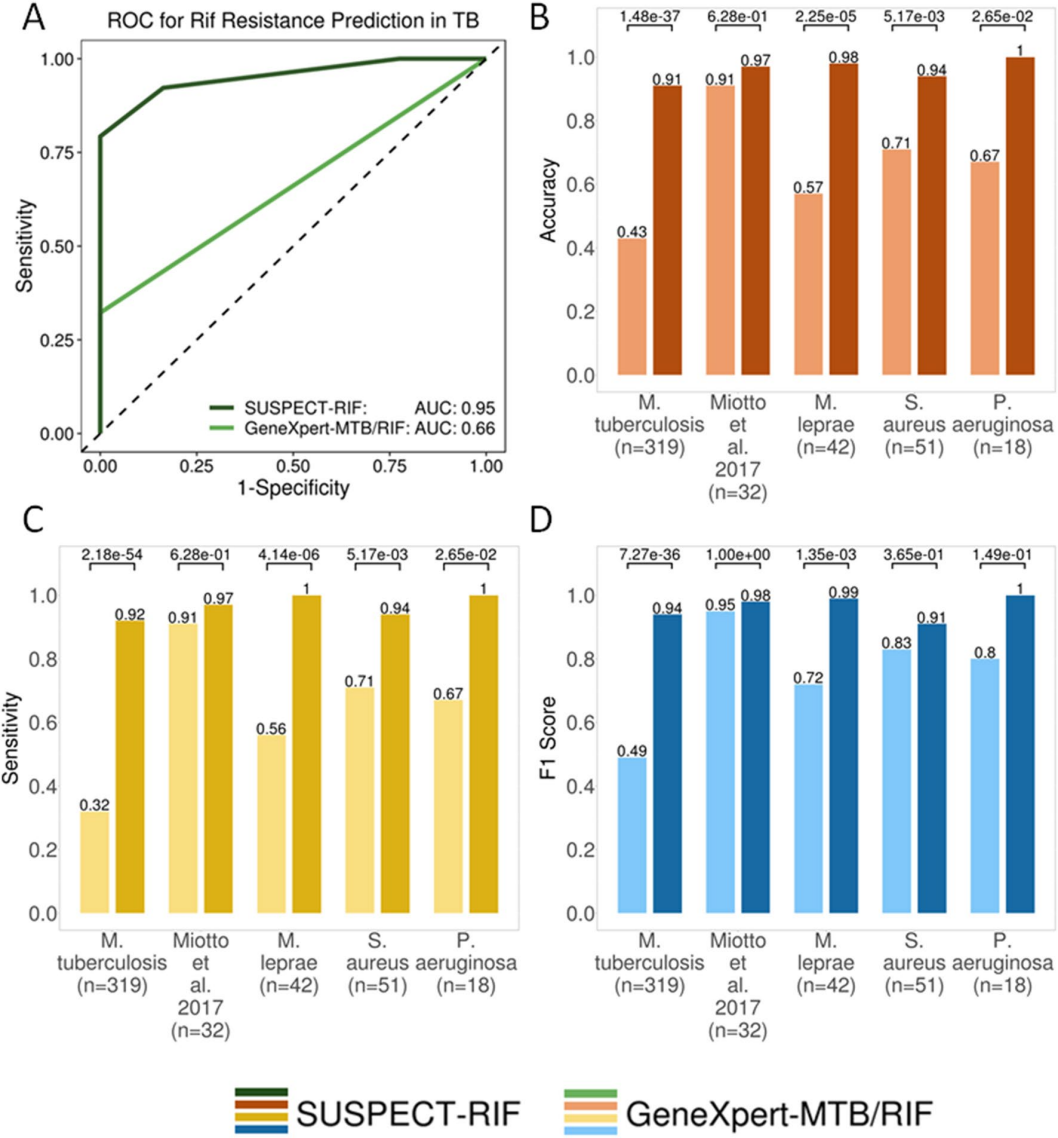
Performance comparison between SUSPECT-RIF and the gold-standard GeneXpert-MTB/RIF. (**A**) The ROC curve shows superior performance of SUSPECT-RIF in successfully distinguishing between RIF-susceptible and resistant mutations on the *M. tuberculosis* dataset (*n* = 319) achieving an AUC of 0.95, significantly outperforming GeneXpert (AUC of 0.66, *p*-value < 2.2E-16). When comparing performance of the two tools across all the different validation datasets, through Accuracy (**B**), Sensitivity (**C**) and F1 Score (**D**) metrics, we show that SUSPECT-RIF significantly outperforms GeneXpert-MTB/RIF across all measures tested, and across all tests. Notably, the highest significance was for the was achieved for the large *M. tuberculosis* (n = 319) test, and the *M. leprae* (n = 42) tests. The least significant results across all metric tested were for Miotto et al.test, primarily because most of these mutations (90.6%) are present within the RRDR, showing comparable performance to the gold standard. As for the *P. aeruginosa* and *S. aureus* mutational sets, lower significance values across the metrics, leading to non-significance when considering F1 Score, is thought to be a direct result of sample size and proportion of mutations in RRDR (66.7% and 70.6% respectively). All significance tests were computed using a two-tailed z-test with continuity correction.

Following greedy feature selection, our trained predictive classifier had comparable MCC between training (0.72) and blind test (0.71), with an AUC (Area Under Precision Recall Curve) of 0.99 and 0.89 respectively (Suppl. Figure 4A). When tested on all mutations in our training and blind *Mtb* datasets (*n* = 319), our initial model’s performance in identifying resistant (sensitivity) and susceptible (specificity) mutations was 92.2% and 81.6%. For mutations located inside the RRDR, the model correctly predicted all resistant mutations (*n* = 87; 38 in training set, 49 in blind test), having an identical performance to GeneXpert-MTB/RIF. Finally, the performance for all *Mtb* mutations outside the RRDR, for which there is no current molecular test, was at a sensitivity of 89.1% and specificity of 89.8%. Based on the assumptions underlying the GeneXpert-MTB/RIF, outside the RRDR it captured 0% of the resistant mutations and 100% of susceptible mutations. The distributions of performance across train and test sets are described in Table 1. Finally, a comparison of our final classifier, SUSPECT-RIF, with GeneXpert-MTB/RIF performance on the full *Mtb* (*n* = 319) dataset shows significant improvement in resistance detection (Fig. 4A; *p*-value < 2.2E-16), suggesting clinical applicability in *Mtb* infections.

## Clinical validation

### Clinical mutations in tuberculosis

To further validate this applicability, we subjected SUSPECT-RIF to the *Mtb* mutations reported in Miotto et al*.*, 2017^51^ (*n* = 32), of which 25 were high confidence resistant mutations, 4 were moderate confidence, and 3 were low confidence mutations. Our classifier correctly predicted the mutations as resistant with 96.9% accuracy and 100% precision, when compared to 90.6% accuracy and equivalent precision for GeneXpert-MTB/RIF (Fig. 4B–D; Suppl. Table 3). Most mutations (*n* = 29) in this dataset were located within the RRDR, which explains comparable performance between the two methods. The only variant misclassified by SUSPECT-RIF was I491F, which was deemed as a low confidence mutation in the Miotto et al. study, showing weak clinical resistance evidence. Structurally, this mutation lies at the interface close to the RpoC, and RNA binding (Suppl. Figure 5), where the introduction of the phenylalanine side chain may affect these inter-molecular interactions differently. Introduction of this larger side chain, however, doesn’t seem to introduce major steric clashes. One possible reason behind this misclassification, which might explain the weak clinical evidence in the Miotto^51^ study, is that this mutation is resistant only in specific lineages, which may not be appropriately captured in our model.

We then compared the performance of SUSPECT-RIF on Rif resistant *Mtb* mutations considered in various genome sequencing techniques and databases (Sanger sequencing, CASTB^57^, KvarQ^58^, Mykrobe^59^, PhyResSE^60^, and TBProfiler^61^) curated by Schleusener et al*.,* 2017^52^. For a small dataset (*n* = 7), detected by Sanger sequencing, our model successfully identified all mutations as resistant. We also carried out an analysis of a larger curated dataset^52^ which combined well-characterized mutations from four listed genomic tools^58–61^ (*n* = 539) compared in the Schleusener et al*.* study. Here, SUSPECT-RIF had the highest resistance detection rate of 99.4%, compared to 95.0% by Mykrobe, 33.0% by KvarQ, 17.3% by TBProfiler and 5.6% by PhyResSE. Of the three undetected mutations, I491F was only detected by PhyResSE and TBProfiler, and deemed low confidence by Miotto et al., further suggesting that it may be strain specific. Mutations T482P and A286V, were only considered resistant by TBProfiler, suggesting that they may also be strain or region specific.

### Translation to leprosy

To test the applicability to other clinically relevant mycobacteria, SUSPECT-RIF was subjected to 42 clinical *M. leprae* mutations curated from the literature^53^, which included a study focusing on a natural leprosy reservoir—the Prata village in the Brazilian Amazon^54^. All resistant mutations were successfully identified (100% Sensitivity) while the one susceptible mutation within the dataset was misclassified as resistant (0% Specificity). The significantly higher performance of SUSPECT-RIF when compared to RRDR-based molecular tests (similar to GeneXpert-MTB/RIF for *Mtb*) on our dataset proves that the clinical utility of SUSPECT-RIF, although trained on *Mtb* data, is not limited to tuberculosis infections (Fig. 4B–D; Suppl. Figure 4B; Suppl. Table 3).

### Translation to other infections

Next, we subjected SUSPECT-RIF to resistance mutations identified in two other, clinically diverse organisms where Rif is reserved as a last line treatment: *P. aeruginosa* (*n* = *18*)^55^ which is Gram-negative (SI: 55%) and *S. aureus* (n = 51)^56^ which is Gram-positive (SI: 61%). Despite the relatively low sequence identities compared to *Mtb* RpoB, our model could correctly detect all resistant mutations in *P. aeruginosa* (100% Sensitivity), while only missing three *S. aureus* mutations (94.1% Sensitivity). Notably, two of these *S. aureus* mutations are present at residue 527, which is equivalent to residue 491 for *Mtb.* Misclassification at this residue might therefore be due to misrepresentation of lineages within our model, as discussed previously. These results (Fig. 4B–D; Suppl. Table 3) further demonstrate how using an initial understanding of molecular mechanisms in one organism (*Mtb*) as a basis for classification (SUSPECT-RIF) can be robustly translated to other structurally and pathogenically diverse organisms.

## SUSPECT-RIF: Web server

SUSPECT-RIF has been implemented as an interactive web-server, which is freely available to the clinical and research community at: https://biosig.unimelb.edu.au/suspect_rif/. Our website allows prediction and visualization of missense mutation phenotype, within *M. tuberculosis*, *M. leprae*, *P. aeruginosa* and *S. aureus*. User input requires the missense mutation, given as an XnY code where X is the one-letter-code wildtype residue, n is the residue position according to organism structure numbering and Y is the one-letter-code mutant residue. We have included the FASTA sequences of the different organisms displayed, as these sequences refer to the numberings used in our protein structures. The user input can either be carried out as a single mutation or as a mutation list, with the different outputs shown in Suppl. Figure 6. Single mutational analysis exhibits the phenotype as classified by our model, mutational information regarding local environment, as well as an interactive molecular viewer built using NGL Viewer^62^. Users can compare changes of interatomic interactions across wild-type and mutant structures in the 3D viewer and download it as a PyMOL session. The results from the mutational list input are exhibited as a summarized list of phenotype and local environment values, with an interactive viewer showing all mutations mapped across the RpoB protein. Links are also available for every mutation within the list to be evaluated and visualized separately.

## Discussion

Our earlier work in analysing resistance mutations in TB has shed light towards the complex phenomena underlying resistance to Rif^19^, mainly because of the different interactions occurring at the transcription cleft. Predominantly, Rif resistance mutations in TB disrupt affinities between the target RpoB and other RNA polymerase subunits, as well as nucleic acids within the cleft. When the same methodology was applied across another mycobacterium (*M. leprae*) and a Gram-negative rod (*P. aeruginosa*), similar mechanistic effects of resistance were delineated. Within the context of the RNA polymerase complex, predominant disruptions at the interface affecting protein–protein and protein-nucleic acid interactions may lead to a lower steric effect imparted by Rif at the structural level. This effect might be overcome in vivo through compensatory mutations, which have already been reported for *Mtb* within gene *rpoC*^20,21^. At the protein level, such mutations can strengthen the interactions between RpoB, RpoC, and nucleic acids to retain the transcription functioning of the RNA polymerase complex and circumvent the steric and consequential nucleic acid affinity-reducing effect of Rif. As for our analysis on the Gram-positive coccus *S. aureus*, the main mechanistic driver of Rif resistance is a loss of ligand affinity. This is thought to be primarily because most mutations within our dataset occur within 10 Å of Rif binding (with 70.6% occurring within the RRDR). This overall mechanistic pattern may also explain the slightly lower performance of SUSPECT-RIF on *S. aureus* mutations, where features describing protein-nucleic acid affinity are more highly accounted for than those for ligand affinity. Finally, no primary mechanism could be seen upon analysis of susceptible mutations (available for *M. tuberculosis* and *M. leprae*). This gave us a solid understanding to base machine learning principles in building a diagnostic resistance tool.

Here, we introduce our diagnostic tool, SUSPECT-RIF, which does not assume resistance on mutational gene location, but accounts for the differences in underlying molecular mechanisms imparted by resistance mutations compared to susceptible ones. Our tool is built on features describing local mutational environment, interactions, flexibility and conservational effects. We have shown that this combination of features achieves high performance in detecting resistance mutations in an independent clinical *M. tuberculosis* dataset^51^ with a comparable RRDR performance to the current gold standard, but also beyond this region. This is especially important considering that resistant mutations outside this region are currently being missed. Overall, SUSPECT-RIF can correctly classify 90.9% of all *Mtb* mutations within our dataset. We tested the robustness of SUSPECT-RIF in detecting well-characterized *Mtb* mutations from whole genome databases^52^ where it outperformed all databases^58–61^ tested, with an accuracy of 99.4%. When considering protein structural effects, where only coding mutations can be analysed, these metrics are also comparable to a whole genome sequencing-based predictor (accuracy: 95.1%)^63^, which is built on larger datasets. This latter tool possibly also includes variation within non-coding regions (raw data not available for comparison)^63^ which cannot be assessed through our method. Apart from comparable accuracy in *Mtb,* SUSPECT-RIF has proven effective in identifying resistance mutations in RpoB across another mycobacterium (*M. leprae*)*,* but also in Gram-positive (*S. aureus*) and Gram-negative (*P. aeruginosa*) organisms where Rif is clinically used. Notably, its robustness in detecting Rif resistance in leprosy makes it the first, genetic-based test for resistance in *M. leprae*. This, given the WHO Global Leprosy Strategy^64^ is crucial in early detection of Rif resistance and appropriate patient therapy—as it improves rates of survival, as well as minimizes further resistance development. Further to that, its clinical applicability in non-mycobacteria has been achieved in spite of low sequence identity with *Mtb*, making SUSPECT-RIF the first structure-based diagnostic tool which consistently performs across bacterial species. This highlights the power and broad applicability of this approach for predicting resistance from both clinical^65,66^ and drug development perspectives^67–70^.

SUSPECT-RIF relies on an interplay of different features (Suppl. Table 1) describing changes in interaction affinities of RpoB with other proteins, nucleic acids and ligand Rif, as well as changes in stability, flexibility (deformation and fluctuation) and residue level changes in interactions. When analysed on their own, some features offer negligible contribution to the model. However, these were retained as they are thought to work in synergy with other molecular properties. One feature which was not tested in our model was bacterial lineage associated with each mutation. During data curation, different sources of data were used, where lineage information was not always present. Because of this, it is thought that our misclassified mutations were lineage-specific, where the same mutation in different lineages may not always be resistant. To address this, we segregated our datasets according to source, where we have trained our model on lineage representative LSHTM^15^ mutations, and tested with non-redundant, literature-identified datasets. This was our approach in obtaining a general lineage classifier, given the limitation of lineage information. Another feature which was not tested was the Minimum Inhibitory Concentration (MIC), which quantifies the degree of resistance conferred by each mutation. As with lineage information, MIC values were not consistently available for all the mutations used in model development, which may also explain some misclassifications by our model. Finally, although *rpoB* mutations have been shown to have epistatic effects both within RNA polymerase (*rpoC*^20,21^*)* and with another TB-drug target *gyrA*^71,72^, which binds TB second-line drug Ofloxacin, we did not have enough data across our whole mutation list to be able to account for epistasis within our model.

When considering overall model performance, our model was consistently more robust in predicting resistance mutations (higher sensitivity) than susceptible mutations (specificity). Although the identification of susceptible mutations in the clinic enables the confirmation of safe Rif use, it is more important for our classifier to identify resistance mutations as efficiently as possible. Clinically, this high sensitivity in identifying resistance mutations implies that patients infected with a resistant strain are not given Rif unnecessarily, reducing the chance of related toxicities to the patient, quicker access to more effective treatment, and unnecessary costs to the healthcare system.

Our tool, SUSPECT-RIF is freely-available on an interactive website (https://biosig.unimelb.edu.au/suspect_rif), only requiring a list of missense mutations to be analysed by the clinician or researcher. Quick identification of rifampicin resistance, especially in TB and leprosy where it is part of the backbone regimen is crucial for effective treatment, with subsequent avoidance of unnecessary drug toxicities and further resistance development. Resistance-identification using SUSPECT-RIF only depends on the time, cost and availability of genome sequencing techniques, and an internet connection. Further to this, our tool has also been successful in the non-mycobacterial pathogens *S. aureus* and *P. aeruginosa,* where Rif is reserved for MDR cases. Our tool shows robust performance in both organisms, making it translatable to both Gram-positive and Gram-negative clinical infections where Rif is used in poly-resistance. This applicability across diverse, clinically relevant organisms demonstrates the potential significance SUSPECT-RIF has in Rif stewardship efforts. Further to this, the robustness with which SUSPECT-RIF classifies Rif resistant and susceptible mutations provides a good basis for the development of a universal rifampicin resistance predictor.

## Methods

### *M. tuberculosis* mutational dataset

A dataset containing both resistant and susceptible mutations within gene *rpoB* was curated from different sources. For the training set, we obtained mutational information from the London School of Hygiene and Tropical Medicine in-house database. This included resistance mutations (*n* = 203) identified through a genome-wide association study carried out on 6,697 clinical isolates^15^ and (*n* = 28) susceptible mutations. A non-redundant test set was manually curated from online databases TBDreamDB^36^, tbvar^37^ and GMTV^38^. Resistant mutations (*n* = 67) were obtained from all three sources while susceptible mutations were only available from the GMTV database (*n* = 21). Notably, mutations were clustered at, but not restricted to the RRDR, but also spread throughout the gene and protein structure (Suppl. Figure 1B).

### Translation mutational datasets

The ability of our *Mtb-*trained classifier to correctly predict resistance mutations was also tested in other organisms, including *M. leprae, S. aureus* and *P. aeruginosa*, in order to assess its translational capabilities*.* Resistance mutations for these three organisms were obtained from the literature, where sources ranged from clinical isolates in *M. leprae* (*n* = 42)^53,54^, to genome sequencing of resistant colonies in *S. aureus* (*n* = 51)^56^ and an analysis on background variation epistasis on fitness cost in *P. aeruginosa* (*n* = 18)^55^. Notably, all three mutational datasets were comprised of resistance mutations, except for the *M. leprae* dataset which contained 41 resistant^53,54^ and one susceptible mutation^54^.

### *M. tuberculosis *Protein structure

The experimental crystallographic structure of the *M. tuberculosis* RNA Polymerase complex was obtained from the RCSB database under the PDB id: 5UHC^35^. This complex is comprised of 6 chains denoting subunits α1 (*rpoA*), α2 (*rpoA*), β (*rpoB*), β’(*rpoC*), SigA (*rpoD*) and ω (*rpoZ*). Rif is bound at subunit β (*rpoB*), while the transcribed DNA is present at the cleft between subunits β (*rpoB*) and β’(*rpoC*). With a crystal structure resolution of 3.796 Å and R_free_ value of 0.267, the structure was considered of adequate quality as a base for our clinical classifier. Resistance to Rif is brought about by missense mutations localizing on the gene *rpoB,* which codes for the β-subunit. Prior to feature generation, the structure was checked for missing atoms and residues using the Protein Preparation wizard in Prime (Schrodinger suite). A total of 23 missing atoms were inserted across the full *Mtb* RNA polymerase structure, 8 of which were present in the β-subunit (*rpoB*). During the subsequent feature generation stage, all calculations were carried out on the β-subunit to encompass local wild type environment and its respective changes upon introduction of mutations.

### Homology modelling of *M. leprae*, *S. aureus* and *P. aeruginosa* RNA Polymerase

The RNA Polymerase complexes of *M. leprae, S. aureus* and *P. aeruginosa* had not been experimentally determined at the time of study. To accurately test translation of our structure-based classifier, homology models of the whole complex were built on PDB id: 5UHC^35^ as template, using the Advanced Homology Modelling Wizard within Maestro (Schrodinger suite). Due to lower sequence identities with *M. tuberculosis* subunits within *S. aureus* and *P. aeruginosa*, initial sequence alignment was carried out by comparing results from MAFFT-DASH^73^, T-COFFEE^74^ and Clustal-W^75^ (embedded within Maestro). Alignments were manually curated to optimise sequence identity and gap penalties. Initial models were analysed through MolProbity^76,77^ and the embedded protein analysis tools in Maestro. Loop refinement and minimization within Maestro were used as necessary to minimize the number of clashes within different RNA polymerase subunits.

### Feature generation

All features were calculated on the wild-type RNA polymerase β-subunit of the four different organisms. For specific features where the mutant structure was required (Arpeggio), this was generated using MODELLER^78^. A total of 298 features were tested to account for the major biological changes brought about by missense mutations. These were subdivided into five feature classes:

1. Graph-based signatures^49,50^: were calculated to represent the wildtype protein structure, capturing both topology and physicochemical properties of the protein by modelling mutated sites as nodes which are connected by edges at different interatomic distance patterns
2. Local environment: Descriptors of wild-type protein secondary structure prediction (SST^46^, IUPRED and Anchor^47^), relative surface area, residue depth and Phi/Psi angles were calculated to account for local residue environment prior to mutation. The impact of missense mutations on protein stability (mCSM-Stability^26^, DUET^27^, SDM^28^), flexibility (fluctuation, deformation energies—Bio3D^48^), and dynamics (normal mode analysis—ENCoM^30^, DynaMut^29^) were calculated to account for structural changes. Finally, in order to model the effects of mutations on intramolecular interactions, inter-residue contacts on wild-type and mutant structures were calculated using Arpeggio^45^.
3. Interactions: The effect of missense mutations on protein affinities to Rif (mCSM-lig^31^), nucleic acids within the transcription cleft (mCSM-DNA^26^, mCSM-NA^33^) and the other RNA polymerase subunits (mCSM-PPI^26^) was generated using the mCSM-suite of computational tools. Finally, distances to these interacting molecules (Rif, nucleic acids and RNA polymerase subunits), along with distance to Mg^2+^ and Zn^2+^ within the cleft were calculated.
4. Pharmacophore: Changes in number of hydrophobic atoms, hydrogen donors and acceptors and positive and negative charges upon introduction of mutations were calculated.
5. Conservation: Sequence-based scores from SIFT^39,40^, SNAP2^42^, PROVEAN^41^ and ConSurf^43^ were calculated to account for conservational changes. Calculations based on the aaindex^44^ and substitution matrices (PAMs, BLOSUMs) were introduced to account for physicochemical amino acid properties and evolutionary probabilities, respectively.

### Qualitative and statistical analysis

A qualitative analysis was performed on predictions for protomer stability (DUET), ligand affinity, nucleic acid affinity and RNA polymerase complex stability (mCSM-PPI) on all the mutations within the different datasets, in a manner previously described^19^. Briefly, these measurements were compared for every mutation within the dataset and the predominant mechanisms being affected was assigned to each mutation based on the extent of destabilizing effect. Effects were prioritized in order of size: mCSM-lig, mCSM-NA, mCSM-PPI and mCSM-Stability. This was done in order to adequately account for all types of protein-interactions, irrespective of interacting partner size. To statistically identify features which distinguish between the two phenotypes (resistant and susceptible) we also carried out a two-sided Welch sample t-test on the *Mtb* dataset mutations, using a cut-off *p-*value of < 0.05, using the ggsignif package in Rstudio. All remaining comparisons between different proportions, such as the comparison of performances between SUSPECT-RIF and GeneXpert-MTB/RIF, and comparisons of mechanistic effects of resistance between different organisms were carried out using a two-sided ztest with continuity correction, through the prop.test function in Rstudio, with a 0.95 confidence level.

### Machine learning

Machine learning was carried out using the sci-kit learn package on representative classification algorithms: Linear Classifiers (Gaussian, Multinomial and Complement Naïve Bayes, Stochastic Gradient Descent), Decision Tree, Nearest Neighbours (KNN), Support Vector Machines (SVM) and Ensemble Classifiers (Random Forest, Extra Trees, AdaBoost and GradientBoosting). To counteract the imbalance between resistant and susceptible mutations within the training dataset, different levels of oversampling (*n*_*OS*_ = 0 to *n*_*OS*_ = 6) were tested for each algorithm at each stage of the training and optimisation process. Each trained model was subjected to a non-redundant blind test described above. The different graph-based signatures (based on different distance patterns) and substitution matrices generated were initially tested separately to identify any significant performance in the resulting classifier. The best graph-based signature and matrix were subsequently added to the other features for further iteration. All models were analysed and prioritized according to consistency in Matthew’s Correlation Coefficient (MCC) results between training set and test set. The confusion matrix of each model in predicting the phenotype of the independent test set values was also considered, where the number of falsely-predicted values was as low as possible – thereby optimising final model sensitivity, specificity and accuracy at every stage of the process. The nearest neighbour (*k* = 5) algorithm was consistently the best classifier at all levels of oversampling and was chosen for further parameter optimisation (*k* = 1, 3, 5, 7, 9, 11, 13, and 15), of which, the model with *k* = 3 at one level of oversampling consistently gave the best performance and was chosen as the algorithm for SUSPECT-RIF.

### Feature selection

As a final optimization, all the features (*n* = 298) generated using the methods previously described were subjected to a bottom-up greedy feature selection process using sci-kit learn. Prior to this, a manual removal of statistically redundant features was carried out, as this would introduce noise to the prediction of novel mutations. Greedy feature selection initially trains and tests all the features individually. The best feature is retained, and combined with the remaining features individually, to identify the feature which reaches the best performance at each iteration. This process continues until all features are included. Model performance was ranked according to MCC where the best model was again chosen based on MCC consistency between train and blind test, to eliminate the risk of model overfitting leading to biased predictions towards resistance (larger dataset).

### Website

The SUSPECT-RIF server front-end was built on the materialize CSS framework version 1.0.0, while the back-end was built in Python 2.7 via the Flask framework (version 0.12.2). It is hosted on a Linux server running Apache.

## Supplementary information

Supplementary Information.
