# Supplementary material for: Prediction of rifampicin resistance beyond the RRDR using structure-based machine learning approaches
Source: Sci Rep. 2020 Oct 22;10:18120. doi: 10.1038/s41598-020-74648-y (PMC7581776; doi:10.1038/s41598-020-74648-y)
Supplement: Supplementary file 1 — Supplementary Information. [file 41598_2020_74648_MOESM1_ESM.docx]

**Supplementary Material**

Prediction of rifampicin resistance beyond the RRDR using structure-based machine learning approaches

Stephanie Portelli^1,2^, Yoochan Myung^1,2^, Nicholas Furnham^3^, Sundeep Chaitanya Vedithi^4^, Douglas E.V. Pires^2,5^, David B. Ascher^1,2,4^ *.

1. Department of Biochemistry and Molecular Biology, Bio21 Institute, University of Melbourne, Victoria, 3010, Australia
2. Computational Biology and Clinical Informatics, Baker Heart and Diabetes Institute, Melbourne, Victoria, Australia
3. Department of Infection Biology, London School of Hygiene and Tropical Medicine, Keppel Street, London, WC1E 7HT, UK
4. Department of Biochemistry, University of Cambridge, Cambridge, United Kingdom
5. School of Computing and Information Systems, University of Melbourne, Victoria, 3010, Australia

* Corresponding author. **Email:** david.ascher@unimelb.edu.au


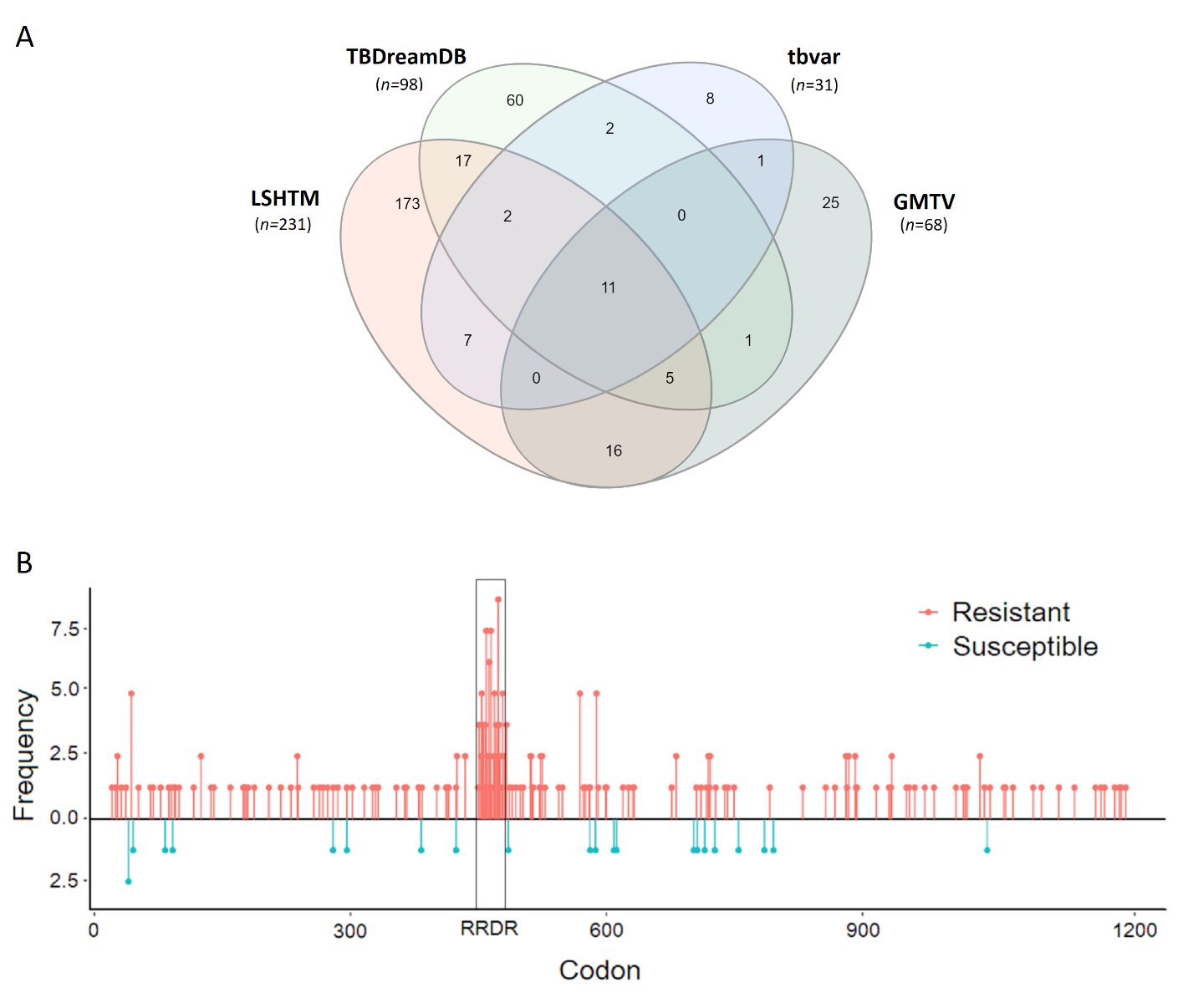


**Suppl. Figure 1.** Distribution of *M. tuberculosis* mutations (A) during the data curation phase, showing overlap in mutations between different sources. In our model, the LSHTM dataset was used as our training set, while the non-redundant blind test was compiled from the other three sources. The distribution of mutations within the training set (B) across the *rpoB* gene, shows ubiquitous distribution of (salmon) and susceptible (cyan) mutations, within and beyond the Rifampicin-Resistance Determining Region (RRDR; black box). No susceptible mutations within our dataset were present within the RRDR.


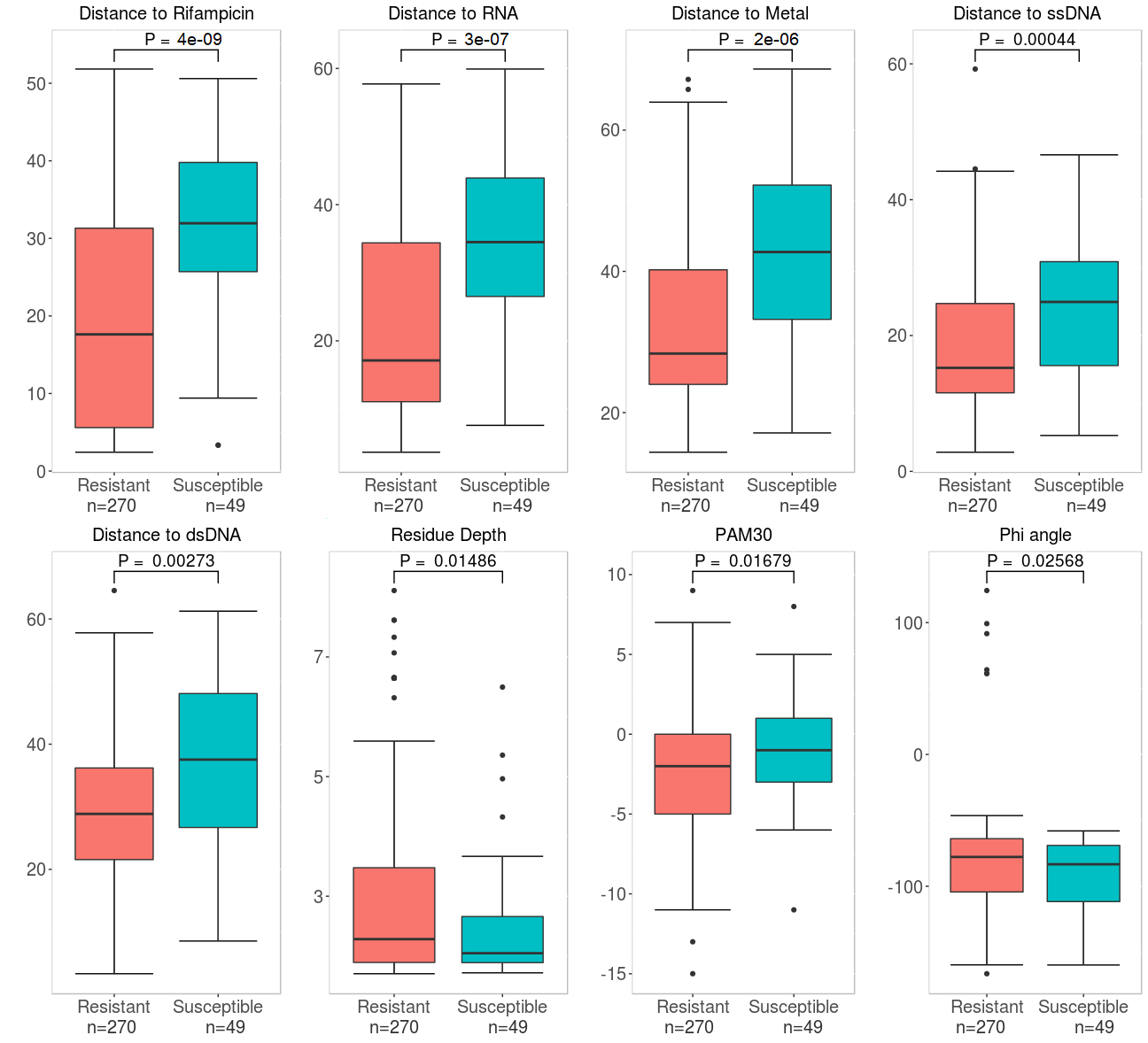


**Suppl. Figure 2.** Additional boxplots for features with good stratification between resistant and susceptible mutations following a Welch sample t-test. Features with the best stratification are described in Figure 3.


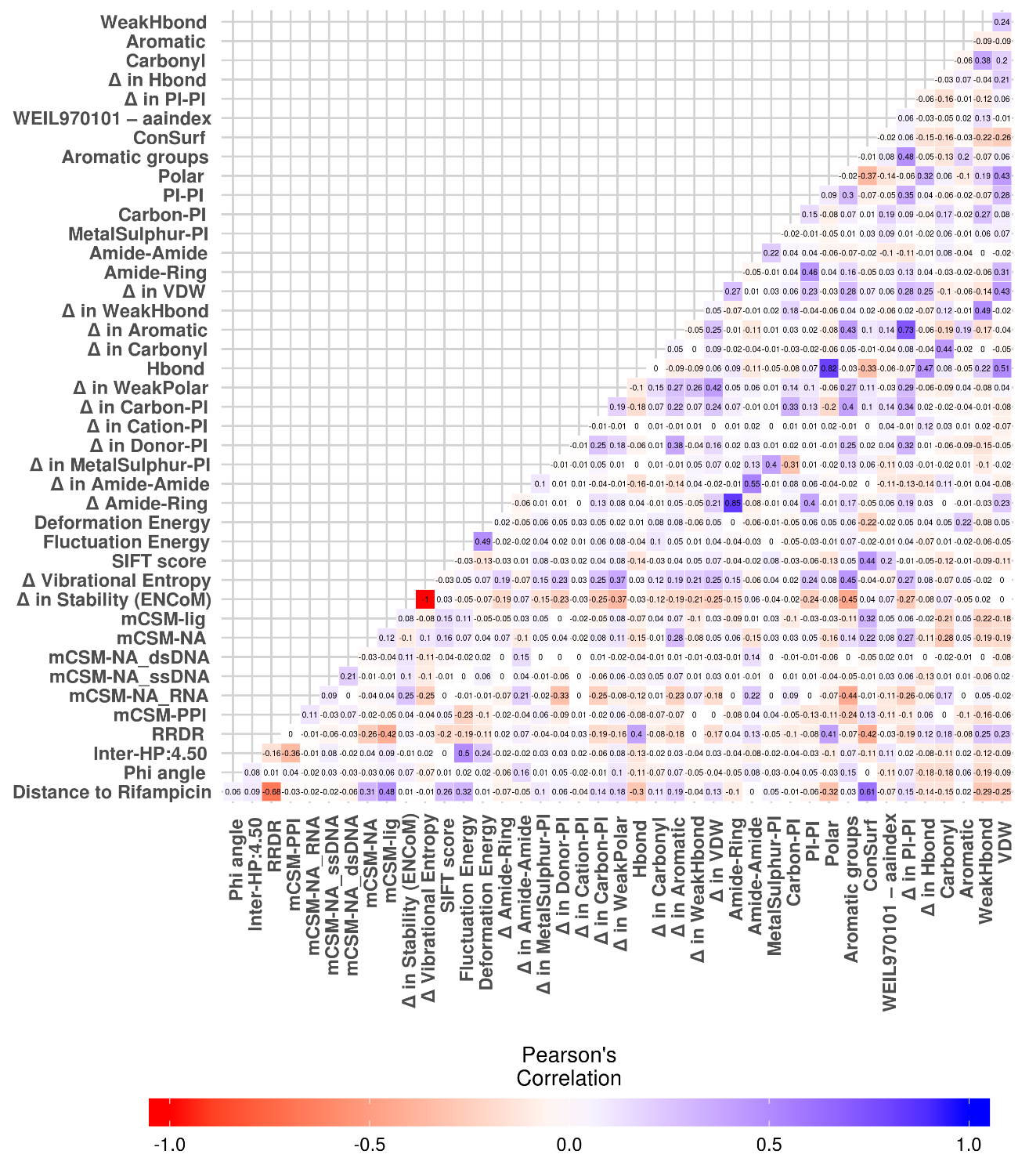


**Suppl. Figure 3.** Correlation matrix of all the features initially chosen by machine learning (n=42), where Δ Vibrational Entropy was removed due to direct correlation with Δ Stability (ENCoM).


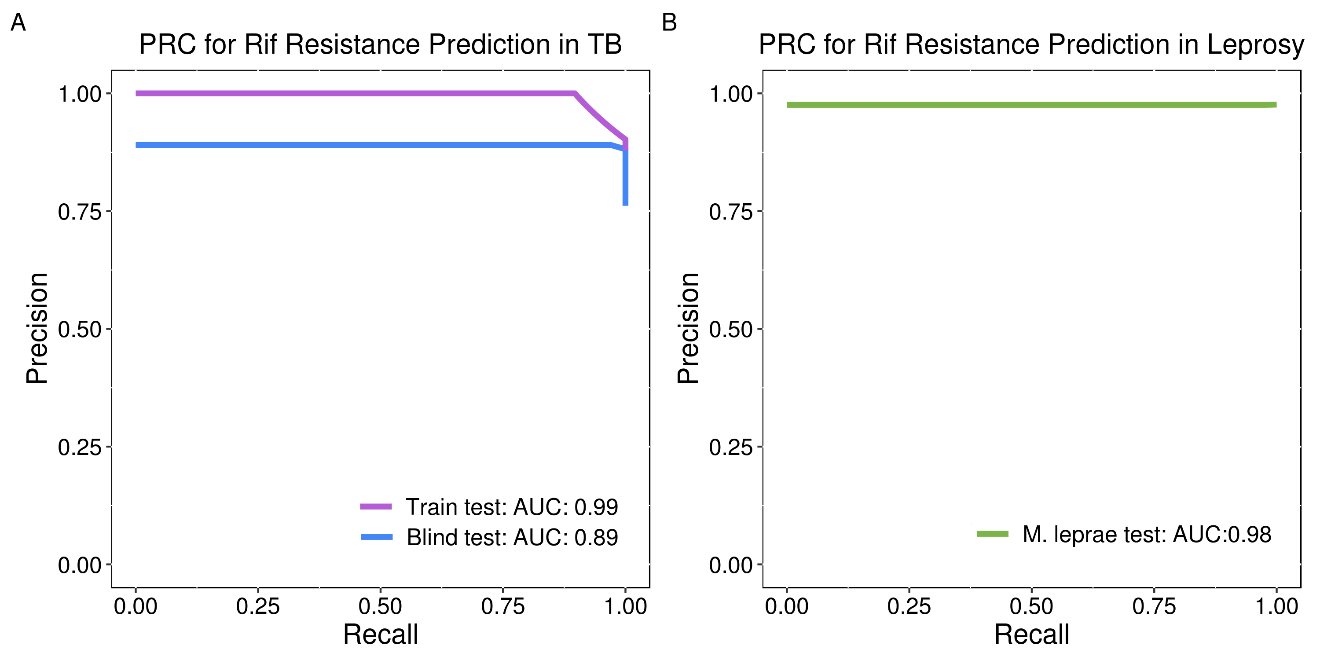


**Suppl. Figure 4.** Precision Recall Curves showing the initial model performance during development. Testing was carried out on (A) *Mtb* training and blind datasets (AUC 0.99 and 0.89 respectively), and (B) on the *M. leprae* test set (AUC 0.98), showing applicability of our model to detect resistance in both mycobacteria.


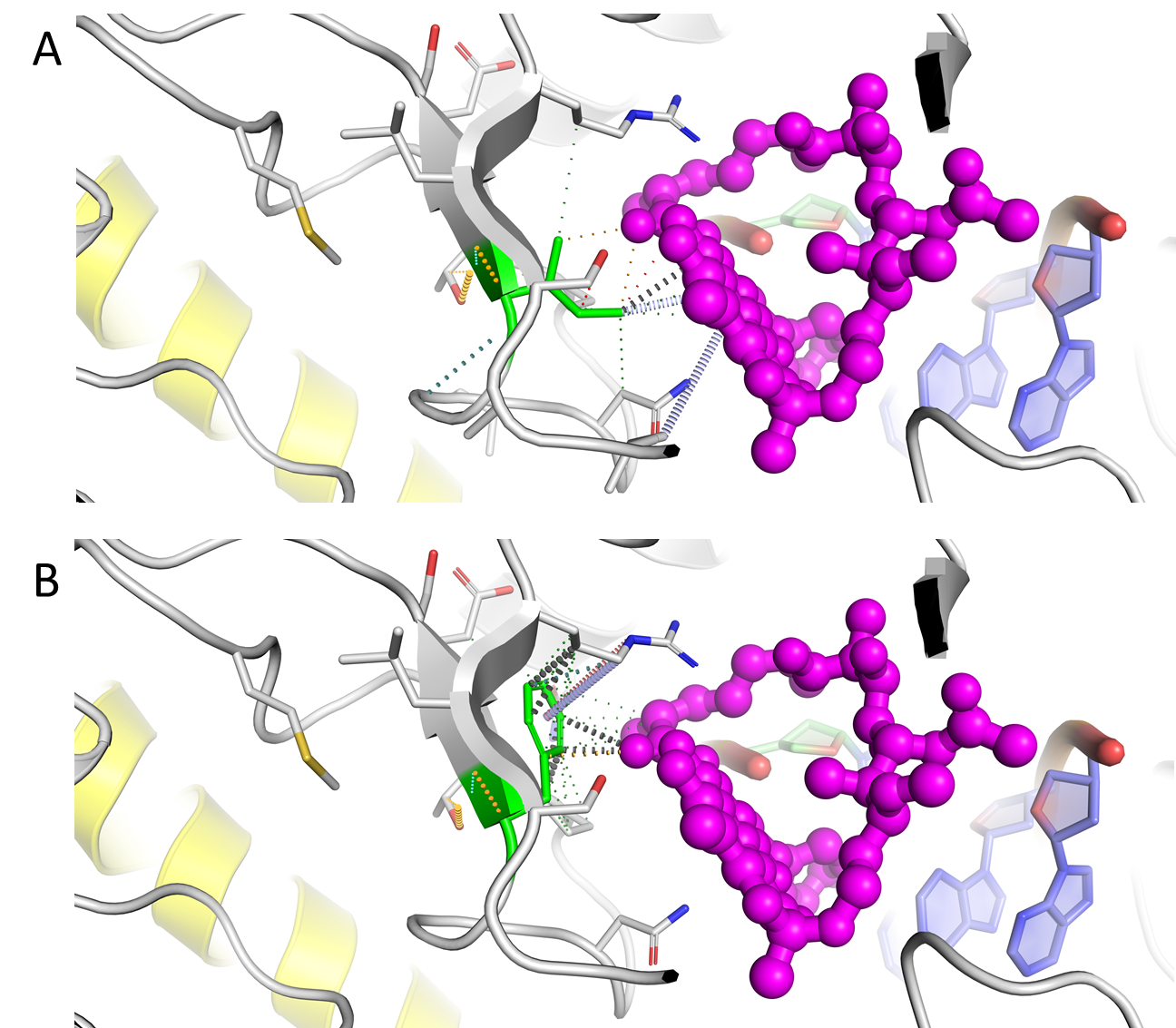


**Suppl. Figure 5.** Interaction networks of different residues at residue position 491. Different networks can be seen between the (A) wildtype Ile and (B) upon mutation to Phe, as seen on PyMOL, downloaded as session files from: [www.biosig.unimelb.edu.au/suspect_rif/run_prediction](http://www.biosig.unimelb.edu.au/suspect_rif/run_prediction). Interactions are coloured according to type: hydrogen bonds in red, hydrophobic interactions in forest, van der Waals interactions in light teal, carbonyl interactions in cyan, polar interactions in bright orange, van der Waals clashes in dark grey, and pi interactions with carbonyl and hydrogen bond donor groups in light blue and salmon respectively. Drug Rif is shown as ball and stick in magenta and interacting protein RpoC is coloured in yellow, showing the mutation’s proximity to the transcript elongation cleft.


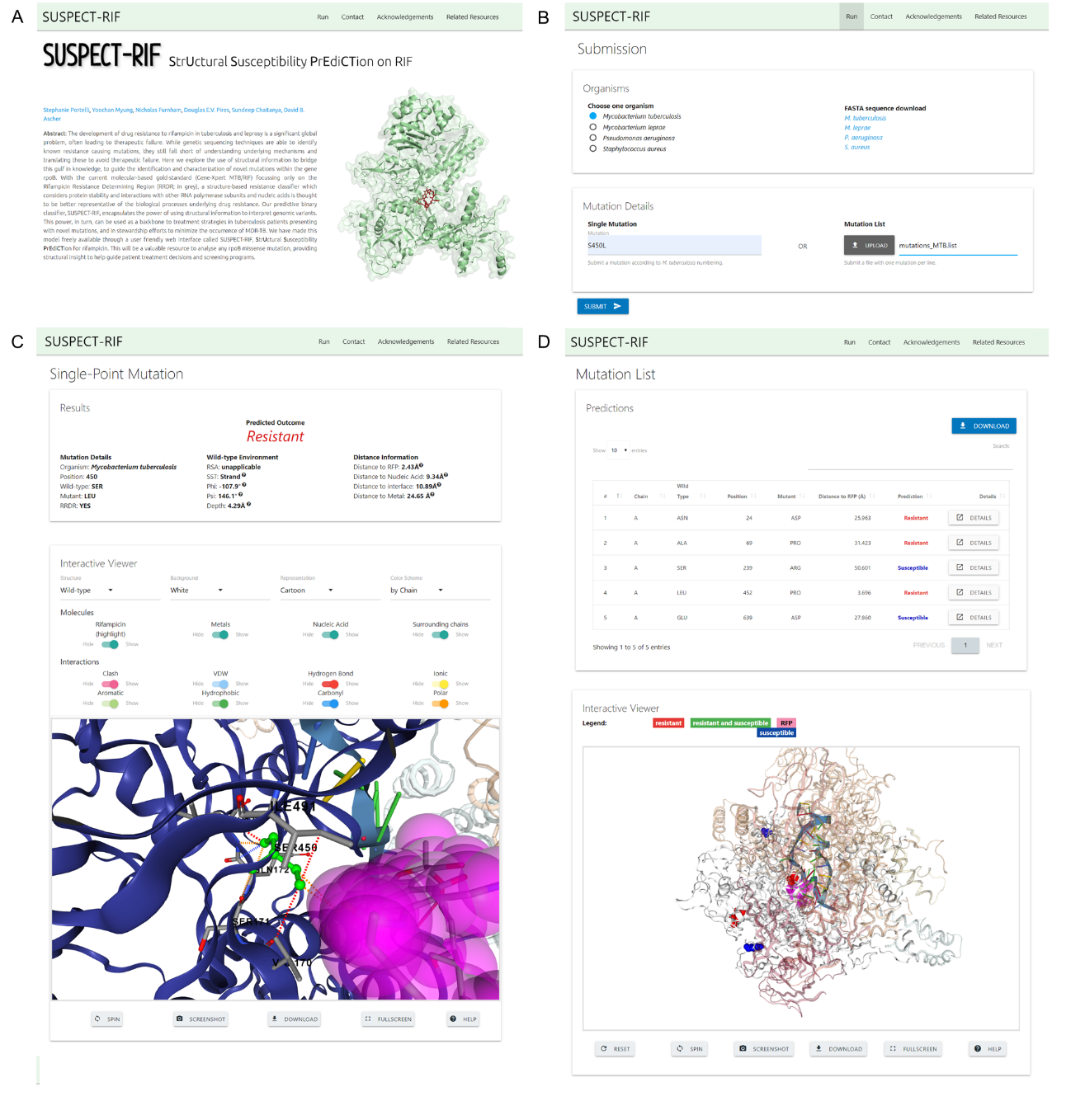


**Suppl. Figure 6.** Visual representation of the SUSPECT-RIF web-interface showing the homepage (A), the ‘Run’ page (B) which initially prompts the user to choose an organism, and enables download of FASTA *rpoB* sequence, and the Results pages, either as single mutation format (C) or as a list (D), depending on the user input.

**Suppl. Table 1.** Statistical Summary of features used in our model following greedy feature selection and removal of redundancies, showing P-values according to Welch sample t-test. All feature classes are represented in the final model, which has a strong representation of local features – helping to recognize differently phenotypic mutations occurring at the same wildtype residue. Features included those calculated on wildtype structure (WT) and those calculated through a difference between wildtype and mutant (∆).

| Feature Class | Feature | Tool | Mean Resistant | Mean Susceptible | ∆ in Means | P-value |
| --- | --- | --- | --- | --- | --- | --- |
| Interactions | Distance to Rif | In-house perl script | 18.94 | 30.93 | -11.99 | 3.86E-09 |
| Local Environment | Phi angle | In-house perl script | -81.60 | -92.27 | 10.67 | 0.03 |
| Graph-based Signature | Inter-HP:4.50 | Graph-based signatures | 1.74 | 0.78 | 0.97 | 0.10 |
| Local Environment | Presence in RRDR | Manual annotation | 0.32 | 0.00 | 0.32 | 1.62E-24 |
| Interactions | ∆ in protein-protein affinity | mCSM-PPI | -0.47 | -0.53 | 0.05 | 0.44 |
| Interactions | ∆ in protein-RNA affinity | mCSM-DNA | 0.22 | 0.93 | -0.71 | 0.05 |
| Interactions | ∆ in protein-ssDNA affinity | mCSM-DNA | -0.06 | 0.16 | -0.22 | 0.15 |
| Interactions | ∆ in protein-dsDNA affinity | mCSM-DNA | -0.27 | 0.04 | -0.31 | 0.13 |
| Interactions | ∆ in protein-NA affinity | mCSM-NA | -0.59 | -0.16 | -0.44 | 0.02 |
| Interactions | ∆ in protein-ligand affinity | mCSM-lig | -0.35 | -0.40 | 0.05 | 0.27 |
| Local Environment | ∆ in protein stability | ENCoM | -0.08 | -0.02 | -0.06 | 0.24 |
| Conservation | SIFT score | SIFT | 0.08 | 0.19 | -0.10 | 0.01 |
| Local Environment | Fluctuation energy | Bio3D | 0.18 | 0.16 | 0.02 | 0.59 |
| Local Environment | Deformation energy | Bio3D | 0.84 | 0.49 | 0.35 | 1.23E-04 |
| Local Environment | ∆ in Amide-Ring interaction count | Arpeggio | 0.01 | 0.02 | -0.01 | 0.81 |
| Local Environment | ∆ in Amide-Amide interaction count | Arpeggio | 0.03 | 0.04 | -0.01 | 0.76 |
| Local Environment | ∆ in MetalSulphur-PI interaction count | Arpeggio | -0.01 | -0.06 | 0.05 | 0.32 |
| Local Environment | ∆ in Donor-PI interaction count | Arpeggio | -0.02 | -0.02 | 0.00 | 0.94 |
| Local Environment | ∆ in Cation-PI interaction count | Arpeggio | 0.00 | 0.00 | 0.00 | 0.32 |
| Local Environment | ∆ in Carbon-PI interaction count | Arpeggio | -0.10 | 0.00 | -0.10 | 0.05 |
| Local Environment | ∆ in WeakPolar interaction count | Arpeggio | -0.23 | 0.08 | -0.31 | 0.16 |
| Local Environment | WT Hbond interaction count | Arpeggio | 2.03 | 1.53 | 0.50 | 0.02 |
| Local Environment | ∆ in Carbonyl interaction count | Arpeggio | 0.05 | -0.02 | 0.07 | 0.17 |
| Local Environment | ∆ in Aromatic interaction count | Arpeggio | -0.29 | -0.16 | -0.13 | 0.40 |
| Local Environment | ∆ in WeakHbond interaction count | Arpeggio | 0.11 | 0.29 | -0.17 | 0.41 |
| Local Environment | ∆ in VDW interaction count | Arpeggio | -0.09 | -0.39 | 0.30 | 0.23 |
| Local Environment | WT Amide-Ring interaction count | Arpeggio | 0.03 | 0.02 | 0.01 | 0.81 |
| Local Environment | WT Amide-Amide interaction count | Arpeggio | 0.11 | 0.04 | 0.07 | 0.05 |
| Local Environment | WT MetalSulphur-PI interaction count | Arpeggio | 0.01 | 0.00 | 0.01 | 0.16 |
| Local Environment | WT Carbon-PI interaction count | Arpeggio | 0.04 | 0.12 | -0.08 | 0.27 |
| Local Environment | WT PI-PI interaction count | Arpeggio | 0.04 | 0.00 | 0.04 | 0.03 |
| Local Environment | WT Polar interaction count | Arpeggio | 2.87 | 2.16 | 0.71 | 0.01 |
| Pharmacophore | Aromatic group count | In-house perl script | -0.22 | -0.45 | 0.23 | 0.44 |
| Conservation | Rate of Evolution | ConSurf | -0.23 | 0.43 | -0.66 | 5.14E-05 |
| Conservation | WEIL970101 | aaindex | -0.18 | -0.06 | -0.12 | 0.46 |
| Local Environment | ∆ in PI-PI interaction count | Arpeggio | -0.11 | -0.12 | 0.01 | 0.89 |
| Local Environment | ∆ in Hbond interaction count | Arpeggio | 0.28 | 0.00 | 0.28 | 0.06 |
| Local Environment | WT Carbonyl interaction count | Arpeggio | 0.20 | 0.22 | -0.02 | 0.77 |
| Local Environment | WT Aromatic interaction count | Arpeggio | 0.04 | 0.02 | 0.02 | 0.48 |
| Local Environment | WT WeakHbond interaction count | Arpeggio | 1.49 | 1.47 | 0.02 | 0.92 |
| Local Environment | WT VDW interaction count | Arpeggio | 1.47 | 0.90 | 0.57 | 8.44E-04 |

**Suppl. Table 2.** Feature interpretability analysis, showing Matthews correlation coefficients (MCC) for performance on blind test for feature subsets, grouped together according to probable synergistic effects. Both structure-based (ENCoM, mCSM tools) and sequence-based (SIFT, ConSurf) features contributed to the model to a large extent.

| Feature subset | Features | MCC test | ∆ in Baseline MCC |
| --- | --- | --- | --- |
| Ligand affinity | ∆ in protein-ligand affinity (mCSM-lig), distance to Rif | 0.56 | -0.15 |
| Physicochemical properties | Phi Angle, Aromatic group count | 0.22 | -0.49 |
| Graph-based signature | Inter-HP:4.50 | 0.19 | -0.52 |
| Protein Flexibility and Dynamics | ∆ in protein stability (ENCoM), Fluctuation and Deformation Energies (Bio3D) | 0.17 | -0.54 |
| Protein-Nucleic Acid Affinity | ∆ in protein-nucleic acid affinities (mCSM-NA & all mCSM-DNA results) | 0.15 | -0.56 |
| Protein-Protein interactions | ∆ in protein-protein affinity (mCSM-PPI) | 0.13 | -0.58 |
| Conservation | SIFT score (SIFT), Rate of Evolution (ConSurf), WEIL970101 (aaindex) | 0.1 | -0.61 |
| Intramolecular interactions | WT and ∆ in intramolecular interactions (Arpeggio) | 0.03 | -0.68 |
| Presence in RRDR | RRDR | 0 | -0.71 |

**Suppl. Table 3** Performance comparison between SUSPECT-RIF and the current gold standard – GeneXpert-MTB/RIF, all the different datasets from different organisms used in clinical validation. Numbers show performances throughout the whole gene in all organisms.

|  | SUSPECT-RIF | | GeneXpert-MTB/RIF | |
| --- | --- | --- | --- | --- |
| *Metric* | ***Sensitivity*** | ***Specificity*** | ***Sensitivity*** | ***Specificity*** |
| *M. tuberculosis* (n=319) | 92.2% | 83.7% | 32.2% | 100% |
| Miotto *et al*., 2017 (n=32) | 96.9% | * | 90.6% | * |
| *M. leprae* (n=42) | 100% | 97.6% | 56.1% | 100% |
| *S. aureus* (n=51) | 94.1% | * | 70.6% | * |
| *P. aeruginosa* (n=18) | 100% | * | 66.7% | * |
| *Metric* | ***MCC*** | ***F1-score*** | ***MCC*** | ***F1-score*** |
| *M. tuberculosis* (n=319) | 0.69 | 0.94 | 0.26 | 0.49 |
| Miotto *et al*., 2017 (n=32) | * | 0.98 | * | 0.95 |
| *M. leprae* (n=42) | * | 0.99 | 0.17 | 0.72 |
| *S. aureus* (n=51) | * | 0.91 | * | 0.83 |
| *P. aeruginosa* (n=18) | * | 1.00 | * | 0.80 |
| *Metric* | ***Accuracy*** | ***Precision*** | ***Accuracy*** | ***Precision*** |
| *M. tuberculosis* (n=319) | 90.9% | 96.9% | 42.6% | 100% |
| Miotto *et al*., 2017 (n=32) | 96.9% | 100% | 90.6% | 100% |
| *M. leprae* (n=42) | 97.6% | 97.6% | 57.1% | 100% |
| *S. aureus* (n=51) | 94.1% | 100% | 70.6% | 100% |
| *P. aeruginosa* (n=18) | 100% | 100% | 66.7% | 100% |

*Undeterminable values.
